# Premature and Incomplete Overview of Health Technology Assessments for Flash Glucose Monitoring Leads to Misleading Conclusions

**DOI:** 10.1177/1932296818822513

**Published:** 2018-12-31

**Authors:** Richard Hellmund, Deirdre Blissett, Fleur Levrat-Guillen

**Affiliations:** 1 Abbott Diabetes Care Inc, Alameda, CA, USA; 2 MedTech Economics, Winchester, Hampshire, UK; 3 Abbott Laboratories Ltd, Maidenhead, Berkshire, UK

**Keywords:** health technology assessment, flash glucose monitoring, diabetes, HTA

Recently in the *Journal of Diabetes Science and Technology*, Stueve and Schnell published the original article “Health Technology Assessments for Flash Glucose Monitoring and How to Use Them in Everyday Clinical Practice.”^1^ A review conducted in February 2018 had identified health technology assessments (HTAs) for a flash glucose monitoring system (FreeStyle Libre system, Abbott Diabetes Care). Eight HTAs were identified, four were considered full HTAs (Canary Islands, Norway, France, Catalunya), and the first two were used to conclude that comprehensive HTAs either recommended flash glucose monitoring for a selected subpopulation (Canary Islands) or found insufficient evidence for a recommendation (Norway).

In this brief overview, important issues were overlooked which have a significant bearing on the conclusions. A narrow definition of HTA was used, leading to conclusions dominated by only two assessments. Given methods vary between HTA agencies, reliable conclusions can be drawn only from a broader cross-section of agencies.

The article appears to overlook how some HTA agencies work, relegating some assessments in importance despite being based on a thorough review of the evidence for flash glucose monitoring. The review by Haute Autorité de Santé (HAS) in France led to a rating of ASA III (https://www.has-sante.fr/portail/jcms/c_2657325/fr/freestyle-libre); this rating, or higher, was obtained in only 8 of 70 evaluations in 2017 (https://www.has-sante.fr/rapport/2017/).

In addition, the Medtech Innovation Briefing (MIB) by the National Institute for Health and Care Excellence (NICE) acknowledged the two flash glucose monitoring RCTs (IMPACT and REPLACE) were of good quality https://www.nice.org.uk/advice/mib110. The article didn’t mention that the Norwegian Institute of Public Health (NIPH) is limited to performing evaluations and does not make recommendations.

The article implies that study quality was not investigated if an assessment tool was not used. However, many HTA bodies assess risk of bias by other means and use of an assessment tool relies on reviewers’ judgement. Differing conclusions regarding the validity of RCT results are therefore expected; for example, in cases where it is not possible to blind the intervention.

Analytical issues associated with the NIPH assessment were overlooked (https://nyemetoder.no/Documents/Rapporter/FreeStyle%20Libre%20Flash%20Glucose%20Self%20Monitoring%20System%20A%20Single%20Technology%20Assessment%20Rapport%202017%20V2.pdf). It was inappropriate to conclude that flash glucose monitoring did not appear to provide greater efficacy than SMBG based on a meta-analysis of the two RCTs because of substantial differences between the study populations (uncontrolled T2DM using MDI therapy and well-controlled T1DM using MDI therapy). The impact of the meta-analysis was to markedly increase the width of the confidence interval, obscuring the substantial reduction in hypoglycemia that was observed across multiple endpoints in the individual studies.^2,3^

The article did not mention that the Canary Islands’ HTA was produced in April 2016, before the primary publications of the RCTs were available; therefore, insufficient information was available for the assessment.

We agree with the authors that the article is a relatively brief overview of a complex topic. Unfortunately, this brevity has caused key information from the available HTA reports to be excluded, leading to conclusions which are selective, premature, and do not reflect the range of HTAs for the flash glucose monitoring system.
